# A Two-Way Interactive Text Messaging Application for Low-Income Patients with Chronic Medical Conditions: Design-Thinking Development Approach

**DOI:** 10.2196/11833

**Published:** 2019-05-01

**Authors:** Monika Marko-Holguin, Stephanie Luz Cordel, Benjamin William Van Voorhees, Joshua Fogel, Emily Sykes, Marian Fitzgibbon, Anne Elizabeth Glassgow

**Affiliations:** 1 Department of Pediatrics College of Medicine University of Illinois at Chicago Chicago, IL United States; 2 All Voices Consulting, LLC Phoenix, AZ United States; 3 Department of Business Management Brooklyn College Brooklyn, NY United States

**Keywords:** mobile applications, telemedicine, patient participation, patient acceptance of health care, delivery of health care, family health, community health services, healthcare disparities, information technology, cell phone use

## Abstract

**Background:**

Two-way interactive text messaging between patient and community health workers (CHWs) through mobile phone SMS (short message service) text messaging is a form of digital health that can potentially enhance patient engagement in young adults and families that have a child with chronic medical conditions such as diabetes mellitus, sickle cell disease, and asthma. These patients have complex needs, and a user-centered way can be useful for designing a tool to address their needs.

**Objective:**

The aim of this study was to utilize the user-centered approach of design thinking to develop a two-way interactive communication SMS text messaging tool for communication between patients or caregivers and CHWs.

**Methods:**

We applied a design thinking methodology for development of the SMS text messaging tool. We collected qualitative data from 127 patients/caregivers and 13 CHWs, health care professionals, and experts. In total, 4 iterative phases were used to design the final prototype.

**Results:**

The design thinking process led to the final SMS text messaging tool that was transformed from a one-dimensional, template-driven prototype (phases 1 and 2) into a dynamic, interactive, and individually tailored tool (phases 3 and 4). The individualized components consider social factors that influence patients’ ability to engage such as transportation issues and appointment reminders. SMS text messaging components also include operational factors to support staff such as patient contact lists, SMS text messaging templates, and technology chat support.

**Conclusions:**

Design thinking can develop a tool to meet the engagement needs of patients with complex health care needs and be user-friendly for health care staff.

## Introduction

### Patients with Chronic Medical Conditions

Low-income Medicaid beneficiaries, in particular children and young adults with chronic medical conditions (CMCs), are at greater risk for poor health outcomes as compared with the general population [1-4]. Children and young adults with CMCs typically have one or more chronic health conditions, have extensive health service needs, and often have functional limitations [5,6]. Managing their condition requires strict adherence to their treatment plan, which can be impacted by psychosocial factors including economic barriers. Children and youth with CMCs have a greater reliance on multiple caregivers in their lives and must face a transition to self-management as they grow older and more independent, which can be difficult for many because of moving from a familiar pediatric setting to an unfamiliar adult one [7]. Factors such as fragmented health care services, lack of access to mental health, and other social support services exacerbate CMCs and lead to high utilization of health care resources [8] and overall poor engagement with health care services [9]. Children with CMCs account for 15% to 33% of the total pediatric health care utilization and costs, which is estimated to be about US $50 to US $110 billion annually [5,6,10-12].

### Patient Engagement Using Short Message Service Technology

Patients actively engaged in their health and health care have better health outcomes and lower health care costs [13,14]. For patients with CMCs, high engagement supports greater adherence to their often complex treatment plan, which can prevent additional hospital visits and lower their risk for increased morbidity and mortality [7]. In recent years, digital health, namely electronic health and mobile health (mHealth), has increased in usage and has the potential to provide an enhanced approach to patient engagement in a cost-efficient way through reminders and patient-provider communication supporting treatment adherence and overall health [15,16]. Text messaging through mobile phone short message service (SMS) is a form of digital health that might be used in a low-cost manner to enhance engagement of those with CMCs who have resource-intensive health needs. Currently, texting technology is widely used by those from many cultures, socioeconomic backgrounds, and age groups [17]. Texting technology can reach people in an effortless and low-cost manner. Many low-income families do not have computers, but at least 96% of them have cell phones and 81% of them have unlimited text plans. Texting technology offers an opportunity to engage with these patients in a way that connects to their everyday life [18]. Compared with app software, texting technology does not require a data plan nor the latest cell phone software, which may be difficult for low-income patients to obtain [16]. SMS text messaging has the highest reach with 98% read rates compared with any communication form. Some studies have reported successful implementation of SMS text messaging as a health intervention tool [17,19-21]. Other studies have reported texting as an adjunct tool to enhance health interventions such as in reminders for medication adherence [22-24] and mental health treatment [25] as well as an add-on strategy to reduce the number of severe mental illness episodes in adults [26] and suicide prevention [27]. Texting technology can be characterized as human-based (human-to-human interaction), computer-based (computer-to-human interaction), or hybrid texting (computer sends out bulk messages to participants and a human addresses the replies in a tailored manner) [28].

### Coordinated Health Care for Complex Kids Health Initiative

In an effort to address the health needs of children and young adults with CMCs, the Coordinated Health Care for Complex Kids (CHECK) program began in 2014 [8]. As part of the Medicare and Medicaid Innovation initiative from the Centers of Medicare and Medicaid Services, CHECK focuses on identifying pathways for reducing health care costs, school absenteeism, and promoting engagement of low-income children and young adults with CMCs and their families with their health and care. Participants, ranged from newborn to 25 years, are enrolled in Medicaid (fee-for-service or managed care) and have a diagnosis of asthma, diabetes mellitus, sickle cell disease, or prematurity. A majority of CHECK participants reside in zip codes identified as having a high community need index, a designation based on income, culture, education, health insurance, and housing [29].

To increase engagement and reduce health care costs for low-income patients with CMCs, we developed a hybrid (computer interacting with humans and vice versa) two-way interactive (patient to staff and vice versa) SMS text messaging technology tool in the context of the larger CHECK health initiative utilizing community health workers (CHWs). The combination of mHealth and CHWs aims to address high health care costs by increasing patient engagement and self-efficacy with their health and health care and reducing the number of preventable emergency room visits and hospitalizations [8,30-33]. It also offers an opportunity to identify barriers to patient engagement and support patients in accessing health-related resources that can enhance their overall well-being and ability to tend to their health care needs [34-36]. As patient engagement is a central factor in health care outcomes, it is vital that any tool used to connect with patients be developed with the patient experience in mind [37-40]. Staff operational needs also need to be considered for maximum utilization [39-41]. To ensure that the tool was user-friendly and effective, we utilized design thinking theoretical guidelines [42] for our approach in designing this texting app. The design thinking model is a user-informed design process that takes into account a large amount of user feedback for a bottom-up approach. The focus is on pragmatic, context-dependent, need-based solutions that improve the likelihood of achieving the full potential of an outcome or event. In doing so, design thinking affords a stable method of alternative solutions to redefine the outcome and seamlessly improve upon previous iterations [42].

The purpose of this study was to describe the development of a hybrid texting app to promote patient engagement among low-income Medicaid children and young adults with CMCs. We utilized design thinking, an iterative development process that incorporates feedback at multiple stages, to ensure an effective, relevant prototype. This study has provided a descriptive overview of how to tailor a digital health tool such as SMS text messaging to patients with CMC needs in the context of a CHW-based initiative.

## Methods

### Design Thinking Process

We utilized the design thinking process to guide the development of the SMS text messaging tool [42]. Design thinking is an organic, bottom-up process, which allows the audience to guide and help design the product, hence increasing the likelihood of a successful outcome. There are 5 steps within the design thinking process that can be repeated as the developer gains more information through prototyping and testing [42].

Step 1 focuses on understanding and empathizing with the audience in a holistic way. This allows the designer to immerse with the audience while trying to objectively assess their needs. The designer can then create a product that greatly benefits the users and encourages their commitment beyond product development into successful implementation and ongoing use. Step 2 is to identify the need and define the need-based outcome through a shared viewpoint across multiple stakeholders. To achieve this, the design thinking approach accepts that human behavior is context-dependent and recognizes the importance of developing solutions that match the user’s self-efficacy [43]. Step 3 uses ideation through a multidisciplinary team approach bringing together professional and *lived-experience* experts who are end-users who have real-life experience with the problem that is being targeted by the proposed solution/product [27] to brainstorm on innovative design solutions that meet the need-based outcome. Step 4 is to create one or more prototypes representing the proposed solution. Step 5 is to test the prototype in an attempt to gather end-user feedback for further molding of the prototype toward its full potential to meet the need-based outcome.

The development of the SMS text messaging tool was spread out across 4 phases over a period of 2 years, starting in March 2015 (see Table 1 for details). The design thinking process was adopted for each phase of the development, including a variety of approaches described below, to collect the information necessary to define the users’ needs [44].

**Figure 1:**
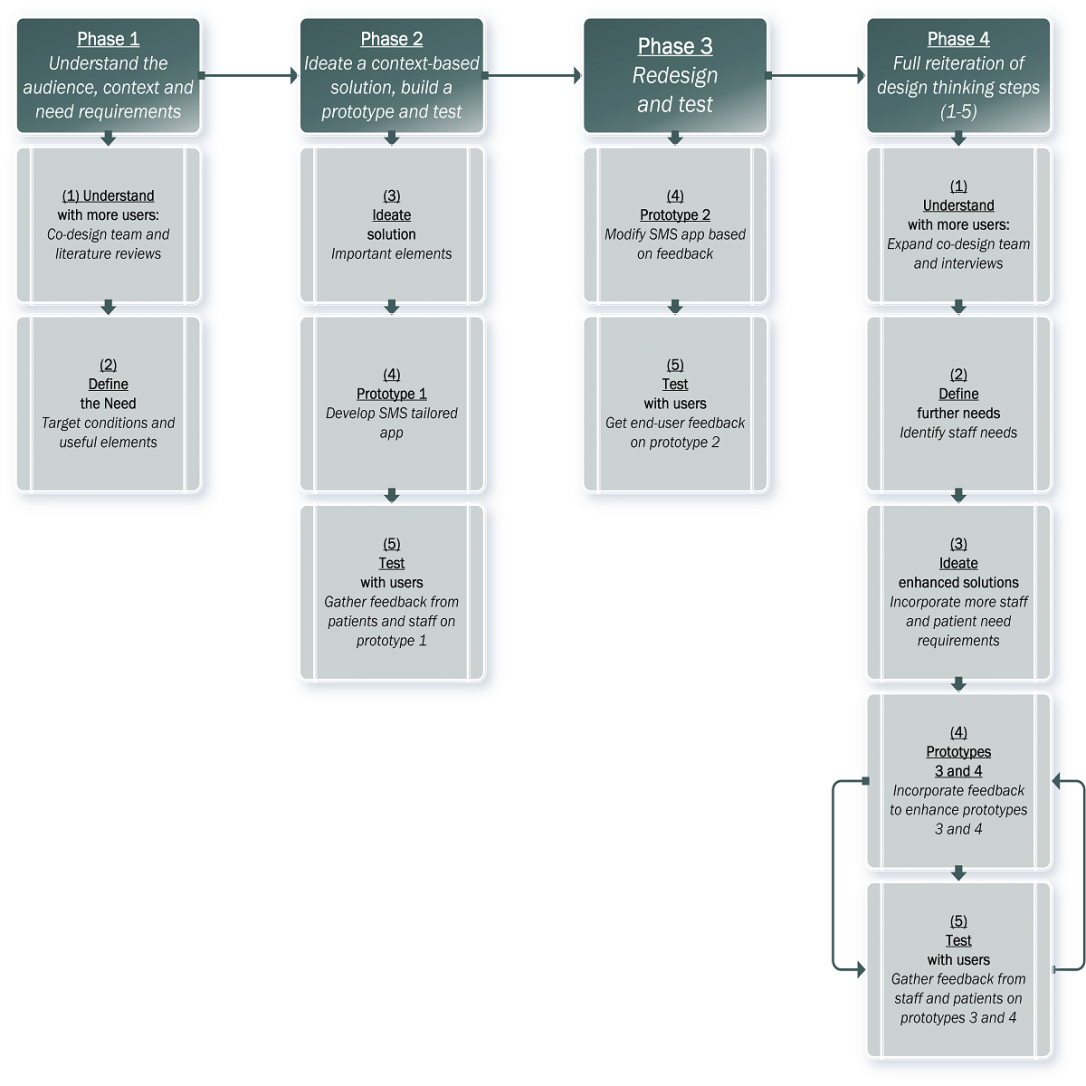
Project phases and design thinking process.

Design thinking encourages the development of rapid and basic prototypes because this permits a more efficient process toward gathering prompt feedback, quickly analyzing it, and only investing in the prototype that would best match the solution under study [42]. We adopted this process across all 4 phases of our tool development and started with an existing texting app (Figure 1).

**Table 1:** Phases of the design thinking process.

Phases and activities		Design thinking steps
**Phase 1 (March to May 2015): Understand the audience, context, and need requirements**		
	Identify our audience as several end-user^a^ groups comprised patients, parents, health care professionals, clinicians, analysts, software engineers, and research designers; create a codesign team of end-users composed of *lived experience*^b^ experts, professionals, and researchers; literature review on SMS^c^ use in health care to understand previous findings about end-users/target population; and literature review on the US population’s broad use of technology-based tools	Understand
	Identify targeted health conditions to determine the set of need requirements with a technology-based tool; and consult with *lived experience* experts, health care, and SMS expert researchers to confirm the need requirements for useful technology-based tools, such as SMS, within the context of health care	Define
**Phase 2 (June to July/August 2015): Ideate a context-based solution, build a prototype, and test**		
	Create an SMS use case^d^ tailored to CHECK^e^ patients with CMCs^f^ and build prototype mockups^g^; ideate with *lived experience* and other expert researchers in a roundtable discussion to evaluate the use case mockups; and build prototype 1 using the outcome results from phase 1 and feedback from the roundtable discussion	Ideate/Prototype I
	Test prototype 1 via a pilot study with CHECK patients to gather patient end-users’ need requirements feedback; and consult with SMS experts and licensed health care professionals on the outcome results	Test
**Phase 3 (August to November 2015): Redesign and test**		
	Modify the SMS prototype solution according to feedback on prototype 1 received by end-users during phase 2 testing; and develop SMS prototype 2 that incorporates the feedback on improving patient end-user engagement from phase 2	Ideate/Prototype II
	Test prototype 2 via a pilot study with CHECK patient end-users to measure their engagement with the tool; and test prototype 2 with a team of health care professional end-users to gather their feedback	Test
**Phase 4 (February 2016 to March 2017): Full reiteration**		
	Expand the codesign team to include software engineers and health data professionals; and conduct individual interviews with CHW^h^ staff to gather need requirements for an SMS tool in the context of health care delivery by CHWs	Understand
	Create prototype 3 to program staff need requirements	Ideate/Prototype III
	Conduct 6 individual interviews to gather requirements from staff as end-users	Test
	Create prototype 4, integrating the patient and program staff need requirements	Ideate/Prototype IV
	Gather feedback through an anonymous survey and 4 individual interviews with staff	Test

^a^Refers to a person who uses or is intended to use the final product.

^b^Refers to end-users who have real-life experience with the problem that is being targeted by the proposed solution/product.

^c^SMS: short message service.

^d^A use case acts as a software-modeling technique that defines the features to be implemented and the resolution of any errors that may be encountered [45].

^e^CHECK: Coordinated Health Care for Complex Kids.

^f^CMC: Chronic medical condition.

^g^Refers to a model or replica of a software model used to provide a visual representation of the model’s app in real life.

^h^CHW: community health worker.

#### Phase 1: Understand the Audience, Context, and Need Requirements

Phase 1, which started in March 2015, was guided by the principles of understand and define in the design thinking process. The objectives of this phase were to identify the end-users; understand their needs for a technology-based solution within the health care context; discover end-users’ need-based requirements; and define the technology-based solution that addresses the needs within the context of health care. We operationalized these objectives into a number of activities, which included instituting a codesign team of end-users, conducting several literature reviews to confirm use of texting in the context of health care for the Medicaid population living with CMCs, as well as determine a set of need requirements through direct feedback from the various groups of end-users.

#### Understanding the End-Users

We identified our audience (end-users) to be comprised several end-user groups (Table 2). The pediatric patients and their parents who formed the Parent Advisory Board (PAB) and their community advocates/leaders, which formed the Community Advisory Board (CAB), were identified as the *lived experience* expert end-users, given their personal experience with living or caring for someone with CMCs [46]. The other end-user groups included the CHECK program staff comprised health care professionals, such as CHWs, licensed clinicians, and health data analysts, and the design team comprised a social scientist, software engineers, a texting research expert, and research designers.

**Table 2:** Summary of end-user groups by phase of the design thinking process.

End-user group	Phase 1, N	Phase 2, N	Phase 3, N	Phase 4, N
Patients/parents	—^a^	95	52	800
Lived experience experts	5	5	—	—
Health care professionals (CHWs)^b^	—	4	3	12
Other health care professionals (operation managers/ supervisors)	—	—	—	2
Licensed clinicians	5	—	—	4
Health data analysts and scientists	—	—	—	6
Software engineers	2	—	—	3
SMS research expert	1	1	1	—
Research Designers	5	5	5	3

^a^Not applicable.

^b^CHWs: community health workers.

We assembled a codesign team of end-users composed of *lived experience* experts, licensed clinicians, software engineers, and researchers. This was informed by previous research highlighting that to address the design requirements of a health care system that aims to meet the goals of both patients and clinicians [47], one needs to have in place an integrated, multidisciplinary design team of professionals [42,48] and *lived experience* experts [27] to successfully execute the design [46].

We also conducted several literature reviews on SMS text messaging use in health care to understand previous findings about end-users/target populations that are difficult to reach (eg, patients aged under 18 years). We used published empirical evidence to inform our understanding of the patient stakeholder group’s needs with their engagement in health and health care [27,46]. This was subsequently followed by a generic literature search and review to understand the US population’s broad uses for technology-based tools [49].

#### Defining the Need Requirements

To begin developing a technology-based solution that satisfied the end-users’ needs, we started by first defining the end-user’s needs. The process of closely refining the definition to meet the end-user’s needs within a given context (ie, health care) is an approach that has led to designing successful outcomes [42,44].

The literature findings on the patient end-users’ need requirements were discussed with the *lived experience* experts, the licensed clinicians, and the research professionals. The discussion resulted in a revised and narrowed definition of the patient end-users’ need requirements for better engagement in their health and care assessed by the use of any technology-based tools. A final targeted literature review was conducted to determine the role and patient use of the technology-based tools within the context of health care [49].

### Phase 2: Ideate a Context-Based Solution, Build a Prototype, and Test

Phase 2, which started in June 2015, was guided by the principles outlining the steps of ideate, prototype, and test in the design thinking process. The objective of this phase was to ideate a use case of the SMS text messaging model app, to demonstrate the texting prototype to the end-users and collect feedback. In this phase, we operationalized the objectives into the activities that included the development of use case mockups that targeted the engagement of CHECK patient end-users with their health and care via a texting app, engaging in a roundtable discussion with the end-user expert groups to gather feedback for the prototype development and test the texting prototype 1 with a group of end-users.

#### Ideate, Develop, and Test Prototype 1

The information gathered through the literature reviews and numerous discussions during Phase 1 converged into the SMS text tool as the technology-based solution to address CHECK patient end-users’ engagement within the context of health care (Table 3). The proposed solution, the texting use case mockups tailored to CHECK patients with CMCs, was presented to the end-user team comprised the *lived experience* and texting research expert, along with other health care professionals. After reading the narrative version of the texting use case templates and reviewing the SMS text messaging app mockups, the end-users engaged in a roundtable discussion with the researchers. The sample questions used during the discussion included “How frequently do you think text messages should be sent out from the provider to the patient?”; “What time of day should a text message be sent out?”; “Is the timing even important and why?”; and “Are text messages that request a response helpful or annoying to you?” The feedback from this roundtable discussion was used to inform the development of the texting prototype app.

**Table 3:** Summary of iterations and prototype outcomes.

Iteration	Ideation	Prototype (patients)	Prototype (staff)	Example
Prototype 1	A two-way SMS^a^ app, non–Health Insurance Portability and Accountability Act (HIPAA) secure, low to no cost, no data or Wi-Fi required, live communication through preset logic to gather insight into patients’ common health-related needs and language that would encourage engagement with their health and care	Explore patients’ language of engagement using 3 methods of engagement: Informative SMS using encouraging and self-motivating text; informative SMS using direct text; generic SMS using neutral text. Explore patients’ needs through 5 common preidentified themes: (1) General information (2) Goal tracking (3) General support (4) Social reinforcement (5) Humor/religious	—^b^	Goal tracking: Do you think you get enough sleep at night? Reply 1=Yes or 2=No; Preset logic for *No* choice: Sleep is important. It is recommended that the average adult gets 7 to 9 hours of sleep each night. Try to make sleep a priority! Preset logic for *Yes* choice: Great! It is recommended that the average adult gets 7 to 9 hours of sleep each night. Keep it up.
Prototype 2	A two-way SMS non–HIPAA secure, low to no cost, no data or Wi-Fi required, live communication through preset logic on 3 themes identified as the patients’ top needs and delayed interactive support by CHW^c^ staff	Explore patients’ level of engagement using text (English and Spanish) to meet their 3 top needs in a delayed two-way interaction with staff: (1) Transportation coordination (2) Social support service delivery (3) Appointment scheduling and reminders	Explore CHWs’ operational needs in addressing and meeting their patients’ top 3 needs in a timely fashion and executed per protocol	Transportation Scheduling: Dear Ms. Doe, the transportation service for your appointment has been scheduled. Your transportation provider is [Fake Transport]. It will pick you up at the address we have on file, on 01/01/01 at 01:00 AM/PM. Please reply *1 or Y* to confirm and keep this service; please reply *2 or N* to cancel it. Thank you, Your CHW; Preset logic for *Yes*: Thank you Ms. Doe; you are all set! We will send you a reminder closer to the date. Your CHW; Preset logic for *No*: Thank you, I will call you shortly to reschedule. Your CHW
Prototype 3	A two-way SMS HIPAA secure, low to no cost, no data or Wi-Fi required, real live communication, and interactive support by CHW staff	To meet patients’ 3 top needs using a live, two-way interaction with staff: (1) Transportation coordination (2) Social support service delivery (3) Appointment scheduling and reminders	To meet on-the-field staff operational needs for live two-way interaction (English and Spanish) with patients, using an editable and smart library of SMS templates to address patients’ top 3 needs; A self-maintaining secure password reset system for staff	See Figure 2
Prototype 4	A two-way SMS HIPAA secure, low to no cost, no data or Wi-Fi required, real live communication, and interactive support by CHW staff	To meet patients’ 3 top needs using a live, two-way interaction with staff: (1) Transportation coordination (2) Social support service delivery (3) Appointment scheduling and reminders; An opt-out and HIPAA disclaimer feature for patients; A self-managed stop and start enrollment feature for patients	To meet patient-centered care support operational needs for live two-way interaction (English and Spanish): CHWs to patients (1:1 and 1:many) and vice versa; staff-to-staff and global texting. An automated grouping feature (based on any combination of patients’ chronic condition, assigned risk, and zip code); An automated scheduling and reminder feature; A tech-support group chat feature triaging staff issues in real time; A self-learning training manual with 24-hour Web access	See Figures 3 and 4

^a^SMS: short message service.

^b^—Not applicable.

^c^CHWs: community health workers.

In July 2015, we developed the first prototype of the texting app. The goal of prototype 1 was to examine the health-related needs of Medicaid patients with CMCs around 5 common themes, as well as to evaluate patient end-users’ engagement via the use of texting. The SMS text messaging contained language based on the 5 common themes resulting from phase 1 consultations (Table 3). The engagement archetypes were adopted from evidence-based techniques used in motivational interviewing [50] and behavioral activation [51].

The 5 themes were framed around the 3 engagement approaches in the following manner: (1) SMS text messages containing motivational language (eg, “Feeling stressed? Exercise is a great way to help! Grab a friend and plan a walk or bike ride today! Reply 1=great idea! Or 2=no thanks”); (2) SMS text messages containing direct language (eg, “Your doctor wants to know if you have problems or side effects from your medicine. Give him or her a call if that happens. Reply 1=Thanks, I’m all set or 2=I need help with that”), and (3) SMS text messages containing neutral/generic language (eg, “Are you eating mindfully? Look, smell, TASTE your food. Savor each bite. Take your time to allow your MIND to take in what you are eating and you will eat less.”) A total of 50 different texting templates were generated for testing with patient end-users.

In August 2015, we launched the testing of the texting prototype 1. The prototype was tested with a group of 127 enrolled CHECK patient end-users randomly selected and comprising 35% of those who met the Medicaid eligibility criteria and were automatically enrolled into CHECK. The final sample size was 95 patients. Those excluded were 14 patients who asked to stop receiving text messages by replying *Stop*, *I don’t want to participate*, or *No thanks* and 18 patients with nonworking phone numbers. The sample was representative of CHECK premature infant patients and children and young adult patients with asthma, diabetes, and sickle cell disease. Each patient participant was randomly assigned to 1 of the 3 patient engagement archetypes and received approximately 3 SMS text messages (each up to 140 characters long) per week, for a period of 4 weeks, for a total of approximately 12 SMS text messages. Data were collected on patient end-user’s engagement level, as measured by the number of SMS replies received per end-user and patient’s health-related needs as measured by the number of SMS replies per end-user on each of the 5 themes.

**Figure 2:**
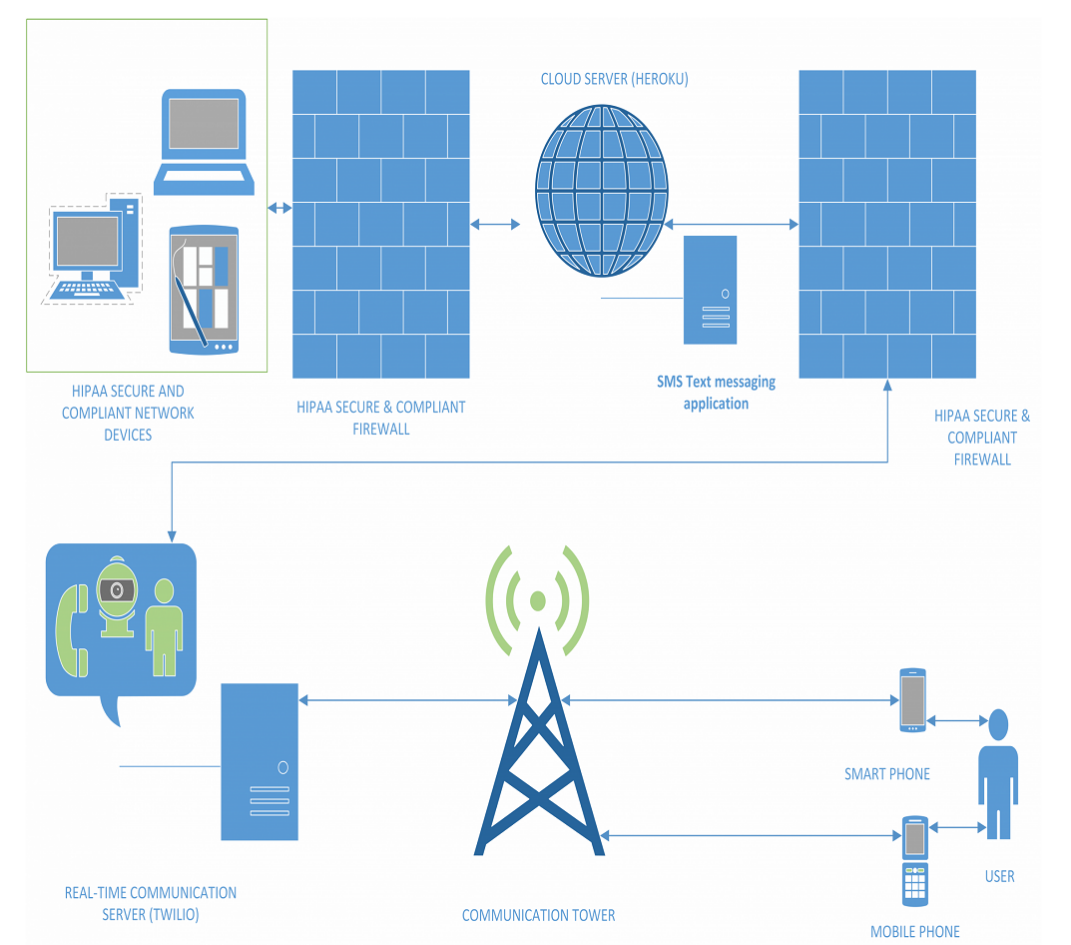
Tool illustration built on an open-source short message service platform (Heroku/Twilio). SMS: short message service.

**Figure 3:**
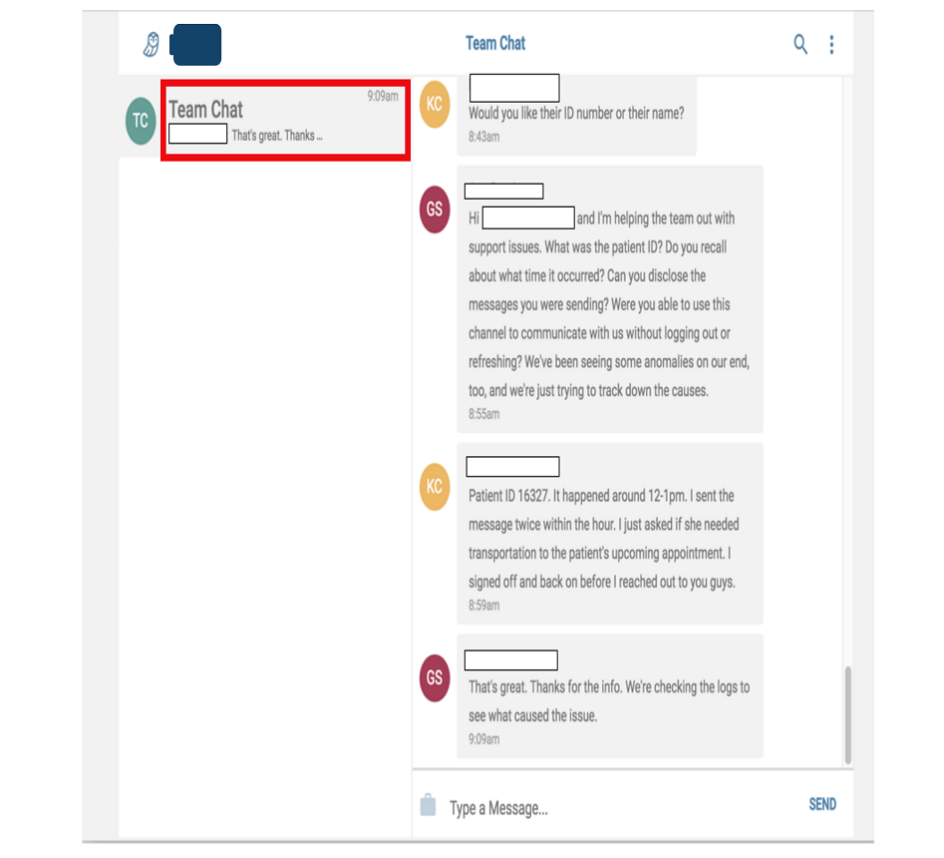
Technology support group chat feature.

**Figure 4:**
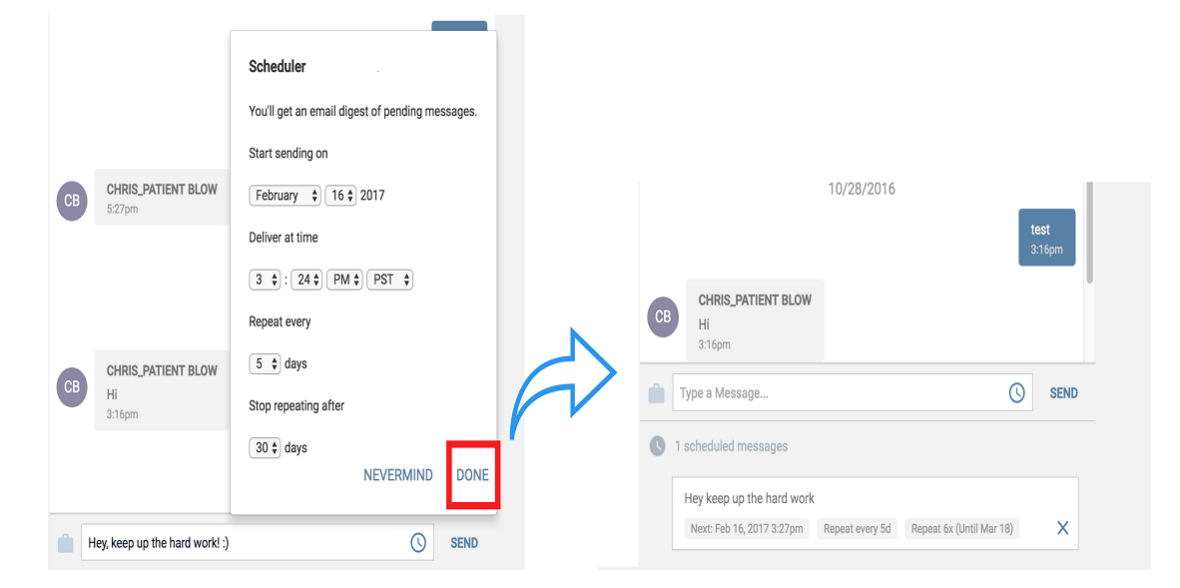
Automated scheduling and reminder feature.

### Phase 3: Redesign Prototype and Test

Phase 3, which started in August 2015, was guided by the principles of redefining the need requirements, prototyping, and testing steps of the design thinking process. The objective of this phase was to ideate, develop, and test iterative versions of the texting app based on newly discovered need requirements from the health care professional (CHW) end-user group. In this phase, we operationalized the objective into activities that included developing the SMS app prototype 2, targeting need requirements from 2 different end-user groups, testing the prototype, and collecting feedback from both end-user groups.

#### Redefining the Need Requirements

The findings from prototype 1 testing were discussed with the CHWs in the form of semistructured interviews (n=4), each lasting approximately 60 min [52]. As the patient’s health care navigators, the CHWs’ feedback exposed a set of operational considerations in addressing their patients’ specific needs in a timely fashion. Additional discussions were held with the texting research expert and the research design team.

#### Ideate, Develop, and Test Prototype 2

The proposed solution for prototype 2 was informed by the patient data collected and analyzed at the end of the second phase as well as the staff feedback. The goal of the second prototype was to address CHW end-user’s need requirements for better patient engagement with the texting tool by targeting specific patient needs in a timely manner. This approach aligned with the self-efficacy principle that emphasizes that individuals’ engagement in certain activities is strongly correlated with their level of efficacy [53] as measured by the outcome of the activity [54]. Prototype 2 included (1) preloaded SMS text messaging reply templates narrowed down to 3 main themes identified by the CHWs as most engaging for patients and (2) preloaded SMS text messaging reply templates that were reconstructed to meet the timing requirements in addressing the patient’s needs.

The second prototype was piloted on 52 CHECK enrolled patient end-users and their respective CHWs (n=3) in October 2015. All 4 CHW staff were instructed to use the tool for a period of 8 weeks with their respective patients needing (1) transportation coordination, (2) social service referral delivery, and (3) appointment scheduling and reminder (all 3 themes identified as most engaging for patients during phase 2). The CHWs were trained to use the Web-based version of the texting prototype tool to send and reply to patient SMS text messages using preloaded SMS text messaging templates centered on these 3 services (Table 3). Patients received at least 1 SMS text message (up to 140 characters long) matching any of the 3 service needs. None of the 52 participants opted out during the testing period. Data were collected on the patient end-user’s engagement level, as measured by the number of SMS replies received per end-user on each of the 3 themes.

Staff interviews conducted during this phase (n=9) were unstructured and were 40 min each [47]. We used unstructured creative interviewing [48] to collect oral reports from our CHW respondents in a more conversational and organic way. Sample questions used during these interviews include “Does the idea of text messaging with patients/caregivers makes sense?”; “Does it make sense for the text message to be integrated into a patient relation management software or electronic health record, or both?”; and “What do you find to be the benefits to connecting with patients via a text message?”

### Phase 4: Full Reiteration

Phase 4, which started in February 2016, involved a full reiteration of the 5 design thinking steps: empathizing, defining, ideating, prototyping, and testing. The objective of this phase was to connect and understand multiple audiences, ideate integrated solutions that addressed the needs of the multiple end-users (patients, staff, and clinicians), and develop and test iterative versions of the texting app prototype that satisfied the final outcome of successfully engaging all end-user groups. In this phase, we operationalized the objective into activities that included identifying and understanding additional new end-user groups; redefining the need requirements; ideating an integrated solution that incorporated the newly defined need requirements as well as developing and testing the texting app prototypes 3 and 4; and collecting feedback from the end-user groups.

#### Identifying and Understanding New End-User Groups

The first 3 phases became instrumental in recognizing the gap of end-user perspectives that led us to expand the codesign team to include not only software engineers and health data professionals but also health care professional support staff (operation managers and supervisors), which we identified as being key leaders to successful implementation and adaptation of the texting app tool by CHWs.

#### Redefining the Need Requirements

The findings from phases 1 to 3 were discussed with the newly added end-user groups through unstructured creative interviews [55], and it was discovered that the first 2 prototypes lacked implementation consideration. This included the delivery of services within the broader health care system and addressed issues such as privacy and security for patient data information exchange and additional reporting needs (see the Results section for specific details). These considerations were used to redefine the need requirements that addressed patient and staff engagement with the tool from this vantage point.

#### Ideate, Develop, and Test Prototype 3

The findings from the first 3 phases and the newly redefined need requirements were operationalized into an integrated solution of the texting tool prototype 3, which encompassed better security, live two-way interaction, and smart support for better triaging (triaging in health care is a concept that deals with the sorting and allocation of services/treatments to patients based on the severity level of their health condition).

A pilot of 10 staff members tested prototype 3 to ensure that its technical aspects met the requirements of the larger health care system for delivery of health care services. The patients’ SMS text messaging activities were not tracked during this testing period. The staff interviews throughout this phase modeled those of phase 3 with the exception of 6 interviews that were preceded by a wireframe SMS text messaging mockup presentation, which was comprised a set of texting prototype tool images that illustrated the working elements of the tool. The interviews during this phase included 4 CHWs, 3 other health care professionals, 1 health data analyst, 1 software engineer, and 1 research designer.

#### Ideate, Develop, and Test Prototype 4

The findings from the prototype 3 testing highlighted the limitation of the patient-centered approach to care, which is a care delivery model whereby patient services/treatments are triaged through a primary care provider ensuring appropriate care at the right time [8]. Prototype 4 attempted to address this need by expanding on key additional features of the SMS tool.

A 23-item survey containing questions about the usability and feasibility of the texting prototype was administered to CHW staff (n=12). The purpose of the survey was to assess the staff’s perception of the texting app final prototype 4. The survey was built and delivered via Qualtrics software that enables researchers to build and deliver Web-based surveys for the purpose of data collection [56]. A sample *yes/no* question was “In the past two weeks have you been using the SMS app?” A sample Likert-type (1=strongly agree to 5=strongly disagree) question was “Thinking back about your experience with the SMS app, please rate the following statement: ‘SMS app is easy to use’.” A sample open-ended question was “What is your least favorite thing about using the SMS app?” The patients’ text messaging activities were not tracked during this testing period.

#### Data Analysis

All the interviews and discussions across the 4 phases were conducted by at least 2 members of the codesign team (1 leading the discussion and the other recording notes). In total, 2 independent researchers, with master’s degrees, transcribed and open-coded the text separately and then openly compared the themes to reach a consensus on the final coding themes [57]. The emerging themes guided the content of the texting use cases and the foundational blocks for the several prototypes built and tested during the third and fourth phase (see Table 1).

The data collected during the pilot studies and the self-administered Web-based staff questionnaire were deidentified, coded, and analyzed using STATA SE, a statistical software package used by social science researchers [58]. The information gathered through all 4 phases was ethically conducted and was approved by the Institutional Review Board at the University of Illinois, Chicago.

## Results

### Phase 1: Understand the Audience, Context, and Need Requirements

#### Identifying and Understanding the End-Users

We identified our end-user groups to be pediatric patients and their parents as well as their community advocates/leaders comprising the *lived experience* expert group; the CHECK program staff comprised CHWs, licensed clinicians, other health care professionals (eg, supervisors) and health data analysts, and a scientist; and the design team comprised software engineers, a texting research expert, and research and social scientists. Further details are shown in Table 2.

To inform our understanding of parents/caregivers and their communities’ needs for better engagement and useful technologies, we used recommendations from members of the CHECK PAB and CAB who served as *lived-experience* advisors and evaluators of the CHECK program components. The importance of recognizing the feedback of those with *lived experience* to guide the design and implementation process of a technology-enhanced tool is in alignment with the patient-clinician-designer framework [44] and has been successfully implemented in one other study [27]. CHWs are trusted members of the community serving as public health workers with a deep understanding of the issues and struggles of their communities [59]. The CHWs’ unique and unusual position as both public health workers and patients of the same health care system allows them to provide innovative solutions to health care–related problems. This approach to problem solving made CHWs an integral part of the multidisciplinary design team. Finally, the CHECK health care professionals whose expertise included clinical social workers, clinical providers, public health, and social scientists helped inform our understanding of the structural and systemic blocks on the benefits and barriers facing technology-based tools in health care [60].

The literature review matching the patients as end-users within the context of health care delivery using SMS text messaging technology was conducted in March 2015. A general search retrieved 911 citations, and 60 relevant studies were reviewed. Patient groups most similar to CHECK’s target population, such as patients with HIV/AIDS (9/60, 15%) and diabetes (8/60, 13%), were identified as the most commonly targeted groups for SMS text messaging studies. The majority of the studies (46/60, 77%) reported improved outcomes. The most commonly reported improved outcomes by SMS text messaging studies focused on improving adherence to medication or treatment (24/46, 52%), increasing appointment attendance (11/46, 24%), and decreasing no-show appointment rates (11/46, 24%). Limitations to using texting within the context of health care centered around privacy concerns with 93% (56/60) of the studies reporting to have remedied this by applying safeguards such as omitting the patient’s name or other Patient Health Information–related information from the body of the SMS text messages. Other limitations cited wrong or invalid mobile numbers because of patients changing them and not reporting the new phone number to the study staff [61] or because of data entry errors [62]. Recommendations for using texting in the context of health care delivery focused on the frequency and themes of the text message content tailored to fit the study’s program goals and the target population.

#### Defining the Need Requirements

The results from the interviews with the *lived experience* experts, professionals, and researchers revealed certain common elements that helped frame the development for a useful SMS text messaging approach applicable to an already existing texting tool. The feedback highlighted the need for a real-time two-way interaction with a feedback loop between patients and providers that was secure but did not require data or a constant internet connection to function properly. Another important point obtained was the need for a tool that highlighted human interaction and noncomputerized intervention programs and placed the focus of communication on the language of engagement and non-disease specific topics, such as goal tracking or social support (see Table 3 prototypes).

### Phase 2: Ideate a Context-Based Solution, Build a Prototype, and Test

#### Ideate, Develop, and Test Prototype 1

The first prototype that was ideated, developed, and tested was a two-way texting tool, non–Health Insurance Portability and Accountability Act (HIPAA) secure, low to no cost, no data or Wi-Fi required, and with live communication through preset logic to gather insight into patients’ common needs and language that would encourage engagement and self-efficacy, particularly with goal setting.

A total of 835 SMS text messages were delivered successfully to 95 respondents over a 4-week period. Of all the sent SMS text messages, 7% (58/835) received responses. The engagement language did not appear to influence whether the patient chose to respond to the SMS text message. The majority of the patients who engaged in SMS replies (14/23, 63%) preferred setting goals and then tracking the goals (7/14, 51%). One patient followed up with the following text reply when prompted to create a goal implying enhanced self-efficacy: “...yeah [that] be so slick like let’s take care of this problem first, I be on the roll.” An overwhelming majority of the respondents (22/23, 99%) preferred SMS text message reminders to keep them on track with their goals.

### Phase 3: Redesign Prototype and Test

#### Ideate, Develop, and Test Prototype 2

The second prototype was designed and developed in response to the patient data collected during Phase 2. Prototype 2 featured a similar app as the first prototype, namely, two-way texting, non-HIPAA secure, low to no cost, no data or Wi-Fi required, and with live communication through preset logic on 3 themes identified as the patients’ top needs. In this prototype, we added a delayed interactive support feature by the CHW staff.

For the testing of prototype 2*,* over the 8-week period, a total of 64 SMS text messages (both computer-to-human and human-to-human) were sent out to 52 unique patients and averaging 6 to 7 patients per week, who exchanged communication regarding their service needs via the texting tool. The overall average response rate (SMS text messages sent-to-received) was 88%. This rate increased to 100% for the categories of transportation coordination and appointment scheduling and reminders.

All 3 CHWs found the prototype compatible with their patients’ needs. Positive feedback included that it “...allows [additional] freedom to operate and customize text as needed” and “The [SMS] web tool is easy to use...with up to 10 patients per week.” The use of the template library was seen as a major benefit to *“*...save [me] time...” and “... stay in compliance [with HIPAA guidelines]...”. The CHWs also reported operational limitations presented by the use of the texting tool prototype. One such limitation was the lack of a universal SMS text messaging phone number similar to a toll-free telephone number. The CHWs found the lack of front-end user interface inconvenient but preferred it as a more comprehensive way of using the tool. A major operational barrier that was reported was having to manually reinsert the patient’s name and phone number for every SMS text messaging interaction, even though this approach ensured that the phone number used to texting the patient was the most recent on file. The unilateral Web-based only platform for use was reported as a major barrier by the CHWs, because it restricted them to manual operations of varying frequency. They felt the need for a more instant approach to monitor and track SMS replies, especially when dealing with at-risk patients.

### Phase 4: Full Reiteration

#### Identifying and Understanding New End-User Groups

As previously described for the texting tool, we identified our stakeholder groups as end-users of various systems within the context of health care. The pediatric patients, their parents, and their community advocates/leaders helped us define the SMS text messaging as a live, two-way interaction with staff for transportation coordination, social support service delivery, and appointment scheduling plus reminders. CHECK program staff comprised CHWs and care coordinators who identified operational needs as an editable and smart library of SMS text messaging templates to address patients’ needs as well as a self-maintaining secure password reset system for staff. During this phase, we expanded our stakeholder audience to include members of yet another important system within the health care context. CHECK professional staff (health care operation managers and health data analysts) helped inform our understanding of the structural and systemic blocks on the benefits and barriers facing technology-based tools in health care.

#### Redefining the Need Requirements

Data collected through informal interviews with these professional staff revealed needs for technology-enhanced staff training, a built-in application programming interface for better integration with existing software cloud-based apps (eg, care management software), built-in dashboards with automated data queries for data exchange, and summary reports with varying levels of security for staff access and supervision. The results pointed to a need for automated feedback. The lack of automatic notification to notify staff of an SMS reply and the delayed SMS text messaging interaction was described as “...very restrictive...not enough [front-end] choices.” Finally, the testing period of the previous 2 prototypes also highlighted the need for diversifying the texting platform to include (1) collecting quality improvement data on the CHECK program as a whole and (2) sending mass notifications to patients about program-funded/organized events with the ability to interact beyond RSVP capabilities.

#### Ideate, Develop, and Test Prototype 3

In February 2016, we redesigned the texting prototype. This redesign addressed the technology-enhanced operational needs and automated feedback feature identified across the stakeholder groups and from the testing phases of the first 2 prototypes. This approach identified a HIPAA disclaimer issue, which was resolved in real time.

The redesigned prototype 3 offered a two-way SMS tool that was HIPAA secure, low to no cost, no data or Wi-Fi required, real live communication, and interactive support by CHW staff. To address the operational needs for better integration with existing software, a troubleshooting feature was implemented. This allowed the CHWs to report their feedback or request technical support via a technology support group chat that would triage the issues to 3 different technology support teams (development, data, and content-based teams).

**Figure 5:**
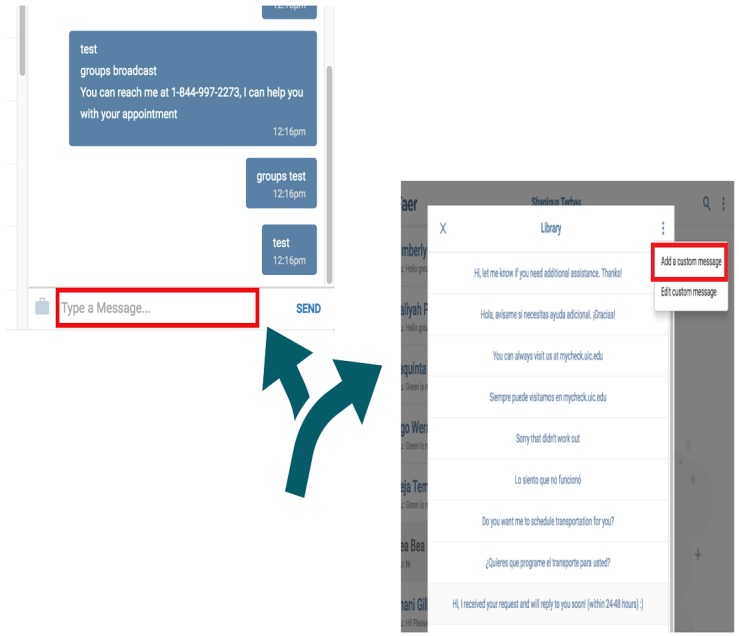
Customizable and self-learning library of text message templates in English and Spanish.

In response to the automated feedback issues, the texting app included a group feature for staff-to-staff communication and also a built-in scheduling reminder texting feature that allowed staff to customize it in terms of frequency and language used to fit the specific needs of the patient (Figure 5).

Furthermore, prototype 3 offered 3 core tools: (1) A secure mode of communicating about the CHECK program and its services; (2) An easy-to-navigate and accessible-from-anywhere information exchange tool that is not restricted by data or Wi-Fi–only devices (eg, smartphone, tablet, or computers); and (3) An instant and interactive mode of information exchange (eg, similar to chat messages; see Table 3 for a full list of features).

On the patient end, there were no operating system limitations. The patients continued to receive the SMS text messages in their native texting app under 1 unique CHECK SMS text messaging number that they were asked to save and use only for texting.

During the testing of prototype 3, the additional need for managing a higher case load and better scalability was further identified by 1 of the CHWs who said:

...with a small number of patients for one or two types of [social] referrals but very time consuming if the number of patients is bigger [than 10] and/or types of referrals expands [beyond transportation and social service delivery]...

Ultimately, the lack of the texting app tool to fully adapt to the identified operational changes by extending the patient-center model of care coordination into the texting app was identified as a barrier that in turn prevented the app’s consistent and uniform implementation by the staff. Full integration of the tool into the care management or electronic health record software was considered to be the biggest limitation across all stakeholder groups.

#### Ideate, Develop, and Test Prototype 4

Prototype 4 took into consideration the major limitation of the patient-centered approach to care. Prototype 4 (see Figure 2) attempted to address this limitation by expanding several key features, such as creating a Web version of the texting app to ensure adoption of the tool by the staff who interacted with the patients only from the office (eg, mental health staff). To partially address the integration issue of prototype 3, we mapped each patient to their assigned CHWs marking the first interoperating bridge between the care management software and the SMS text messaging app tool. To ensure the staff had the most updated patient list, including the most updated phone numbers, addresses, assigned patient risk scores, and chronic conditions, we automated the process to update on a daily basis. Through the use of a proprietary algorithm that worked in the back-end of the data warehouse (to align with HIPAA requirements), we expanded the patient profiles to include the patients’ zip codes, chronic condition, and assigned risk scores to allow patient grouping by service needs for better triaging.

As a result of these changes, prototype 4 featured transportation coordination, social support service delivery, appointment scheduling and reminders, an opt-out and HIPAA disclaimer feature for patients, and a self-managed stop/start enrollment feature for patients. On the field staff side, the case load of patients expanded beyond 200 patients/staff, the prototype provided an automated grouping feature (based on any combination of patients’ chronic condition, assigned risk, and zip codes), a technology support group chat feature triaging staff issues in real time (see Figure 3), a self-learning training manual with 24 hour Web access, and an automated scheduling and reminder feature (see Figure 4).

The final testing for prototype 4 began in January 2017 for both Web and Android versions of the texting app with 4 CHWs servicing 800 patients. The testing period continued for 5 months to allow for all the features to be tested and to rule out any additional technical barriers. The feedback provided was that this final texting app product was appealing and user friendly. More specifically, of the 12 staff surveyed, only 66% (8/12) chose to reply and of them only 4 confirmed use of the texting tool. All 4 CHWs agreed (1/4, 25%) or strongly agreed (3/4, 75%) with the following statement: “SMS App is easy to use”. A similar number also agreed that the layout design of the texting tool was extremely (3/4, 75%) or moderately (1/4, 25%) organized. However, they did not share the same strong sentiment on the overall design of the texting app. When asked, only 25% (1/4) agreed it was extremely good and moderately good (2/4, 50%). One CHW listed the design as neither good nor bad. When asked to further elaborate on why they selected that choice, the answers ranged from “It’s very user friendly” to “it looks bland” to “There is no way to know if the recipient of the text actually received the text.” Regarding the design of the texting app, 3 of the 4 CHWs replied *No* to the question, “Is there anything missing from the SMS App that you were expecting to see (e.g., more text, more images, a FAQ, a question answered, etc.)?” One CHW, who replied in the affirmative, also listed the lack of the feature to allow staff “...to modify patient contact information...” directly in the Web version of the app. Finally, when asked to list their favorite and least favorite things about the texting tool design, 75% (3/4) of the staff listed positive reasons such as “It’s one touch access and it loads ... all the information at my fingertips” and less positive ones such as “Not knowing if the recipient actually received the text” (2/4, 50%). The concern about SMS text message receipt was also highlighted as the main technical issue by 75% (3/4) of the staff members with 25% (1/4) listing “cannot easily reset password” as the only technical issue. When asked to provide additional feedback for further improvement, the staff overwhelmingly (3/4, 75%) stated no feedback for further improvement. One CHW reported “...is a good application. I can’t think of anything else to improve the application”. When asked to list all the barriers that prevented them from using the texting tool, 25% (1/4) checked “Do not feel comfortable with technology”, 25% (1/4) checked “I prefer to use other tools better”, 25% (1/4) checked technical issues with the texting tool, and 25% (1/4) preferred to use “google voice chat.”

#### Natural Language Understanding and Common Responses

Different approaches for natural language understanding were used depending on the prototype. In phase 2, the first 2 prototypes were built with preset branching logic (drawing from common expected replies) and if the reply from the patient fell outside of the predefined range it was automatically forwarded to the staff on call. The staff would then address the answer according to the protocol in place. This was primarily done to ensure the safety of the patients; hence, no automatic SMS reply was sent back to the patient. On the contrary, the staff member (CHW) on call was instructed to reply back to the patient with a tailored answer appropriate to the situation at hand either immediately (if urgent) or within 24 to 48 hours (if nonurgent). Prototypes 3 and 4 automatized this feature to include an SMS text message with a disclaimer about urgent matters and provided a list of emergency phone numbers tailored to the patients’ zip code on file. Furthermore, the last prototype of the tool ensured that the CHW assigned to the patient was notified via text and email. Common responses such as *thanks* or *this wasn’t helpful* were designed to be responded to with a preset branching logic to address common and expected replies. Replies falling outside that range were forwarded to the staff to be addressed within 24 to 48 hours, depending on the urgency of the situation.

## Discussion

### Principal Findings

In this study, we have described the user-informed development and design of a HIPAA secure, two-way interactive SMS text messaging app, serving as the primary mode for information exchange between patients and CHWs. We utilized the design thinking process to take into account multiple perspectives including those of young adults and caregivers to children with chronic health conditions and CHWs in the designing of the tool. The design thinking process centering these users resulted in the development of 4 prototypes leading to a final tool that addressed the needs and barriers during each phase of testing. The final prototype took into account both patient and health care staff preferences to have a tool that is useful for both groups.

The final prototype addressed and included what the patients articulated throughout the testing of other prototypes. The majority preferred texting reminders for tracking goals and appreciated the support in setting them as well. They also wanted the tool to be able to provide appointment scheduling as well as reminders for them. They also emphasized the importance of personalized messaging, human connection, and real time with two-way interaction. Given that the patients were Medicaid recipients, they identified need-based services such as transportation coordination and social support service delivery as helpful to receive through the tool. Finally, HIPAA security was prioritized, given the patient sensitive information exchanged through the tool.

The final prototype also accommodates the changes and preferences of staff feedback given through the testing of other prototypes. For the staff, the tool can be used on the Web and a smartphone/tablet for unlimited access from the office or the field. Assigned patient queues (similar to a contact list in your phone) offer staff the ability to handle a 200+ patient case load. The patient list allows grouping based on factors such as diagnosis and zip code, among others. There is a customizable library of SMS text messaging templates in English and Spanish and an automated patient grouping feature for tailored and efficient mass texting with the ability to receive one-on-one replies. An automated scheduling and reminder feature that is customized and tailored individually for content and frequency ensures that no patient is forgotten. A technology support group chat feature allows for triaging staff issues in real time. This SMS text messaging app has the potential to improve patient engagement with their health and health care.

### Limitations and Future Research

There are several study limitations. First, when working to understand the range of stakeholder views to inform the technology development, we were unable to gather direct user feedback from adolescent patients to inform the codesign team on their perspective. Second, we did not analyze data categorizing the participant texting actual content, response sentiment (eg, positive, negative, and neutral), response frequency (ie, how often a user responded), or response time. Future research should examine these variables to better tailor the tool for end-users. Third, although both patients and staff alike found the tool to be less effective because it was not fully integrated within existing care management technology, the design thinking for tool development did not expand on the established software apps already in use by the program staff. We found that within the context of the larger goals of the CHECK study, emphasis was typically placed on ease of navigation and issues with technical glitches. The integration of this new SMS text messaging tool within existing technology turned out to be extremely challenging because of unprecedented institutional and technological barriers that fall beyond the scope of this study. Despite these barriers, the internal anonymous feedback collected from staff evaluated the tool positively. Fourth, the stakeholder feedback collected from patients’ caregivers and CHECK staff occurred independent of each other. Thus, there was no all-inclusive, connected conversation to simultaneously gauge stakeholder interest from each group. This was intended to be sensitive to the patient population’s needs [27] where such a crossover may create a more intimidating environment or reduce patients’ openness to discuss their experiences with existing services. Fifth, design thinking was the primary framework used to develop the tool, which did not take into account other considerations such as technology acceptance, behavior change, or patient engagement models. Sixth, we were not able to implement a free-to-end-user mechanism for our SMS text messaging tool. Seventh, in adapting the design thinking process to develop the prototypes sequentially instead of in parallel, we limited the product optimization by concentrating on 1 solution. It is possible that sequential prototyping is not as effective as parallel prototyping [63]. Finally, in alignment with the design thinking step 1 of understanding and empathizing with the audience, we put the primary focus on ensuring all stakeholders felt a part of the process and opened up about their experiences without feeling judged. During all phases, we highlighted the importance of anonymity for this purpose and thus did not collect the demographic information of the participants. A strength of this study is the scalability where, in prototype 4, we had 4 CHWs service 800 patients (ie, 200 patients per CHW).

There are a number of areas for future research. First, future research should study if our approach of 1 CHW per approximately 200 patients would work with a larger number of total patients such as 5000 or 10,000 patients. Second, future research also should study if when working with a larger number of patients whether the CHW work load can be increased to more than 200 patients per CHW. Third, in addition to the focus of this study on the process of tool design, it would be useful in future research to determine how this tool or another similar tool would address and potentially positively impact health disparities and social determinants of health. Fourth, with an SMS text messaging tool, there can be many additional ways to tailor content. The final version of the texting tool was developed to meet patient needs on an individual and personalized level. We did not tailor messages based on variables such as diagnosis, response tone, or response frequency as these needs were all addressed on an individual level with personalized texting rather than with large bulk SMS text messages to a larger group of patients. Future research with other SMS text messaging tools that emphasize bulk SMS text messages should consider other areas of tailoring such as tailoring based on disease type, length of sickness, response tone, preferred frequency, and time of day. Finally, with a typical texting tool, it is important to track the *opt-in/opt-out* of patient participants. We did not track this measure for the text messaging tool on the latter prototypes as we relied on the opt-out measure for the CHECK program. Future research should track both measures to accurately measure end-user differences on program preferences versus SMS text messaging tool preferences.

### Comparison With Previous Work

In alignment with a previous study on SMS text messaging tool use [27], our stakeholder groups asked for the use of a mobile phone texting feature as a contact method. This may be due to a steady shift in recent years by most health care systems in using one-way texting to confirm or remind patients about their doctor visits. There is also previous research that utilized messaging platform features to improve engagement specifically among Medicaid patients, confirming that texting can be an effective way of reaching low-income populations in particular [64].

In terms of the patient experience, the design thinking process is similar to research highlighting the need to address the barriers with health interventions [36]. Our SMS text messaging tool was adapted to patient-reported needs and included resources to address barriers to engagement and health care access such as transportation coordination and social support service. The final SMS text messaging app product extends the existing texting prototypes [17,27] through the inclusion of dynamic features for a more *real-life* information exchange experience that can promote patient self-efficacy and meet the needs of both patients and health care staff. Similar to previous research where female adolescent patients expressed privacy concerns with texting [65], interviews with stakeholder groups suggested a need for an SMS text messaging tool that remains compliant with HIPAA standards of patient privacy. This concern was addressed in real time during the third phase of the protocol by including privacy protections to ensure all patient communication was HIPAA compliant.

In terms of the staff experience, a major barrier noted by providers in phase 3 was the re-entry of patient name and phone number with each message to ensure that the patient phone number was the most recent on file. Previous research suggests inaccurate phone numbers are a significant concern to some providers and manual re-entry may be 1 way to address this concern [66]. Although manual re-entry was found to be a barrier to use by the professional staff in our project, it could prove to be valuable in addressing the concerns surrounding inaccurate phone numbers by ensuring that the most recent phone number on file is used.

### Conclusions

The design thinking process was particularly important for the context of this tool because of the need to accommodate varying users, namely, young adults and caregivers of children with chronic health conditions, along with CHWs. Each group had its own preferences and for the tool to be utilized in an effective way, it needed to incorporate the essential preferences of all. Our hybrid computer-human two-way interactive SMS text messaging tool may be especially useful to patients who are unable, because of a lack of resources such as time, data, and Wi-Fi, to interact face-to-face with their health care team. We recommend that when developing a technology-based interactive tool, developers use a validated approach such as the design thinking process to ensure that the final product is compatible with the needs of the target population. It is also important that product evaluation needs be ongoing to address the stream of information from multiple systems of stakeholders. Finally, additional focus should be placed on hybrid computer-human interactions that utilize existing low-cost and easy-to-use technology, such as SMS text messaging, to optimize personalized experience and the likelihood of enhanced patient engagement. Future research would be useful to study if the texting tool works with other patient age groups and patient-centered care teams of professional staff.

## Abbreviations

CAB: Community Advisory Board
CHECK: Coordinated Health Care for Complex Kids
CHW: community health worker
CMC: chronic medical condition
HIPAA: Health Insurance Portability and Accountability Act
mHealth: mobile health
PAB: Parent Advisory Board
SMS: short message service
